# Development and validation of an interpretable machine learning model for predicting 5-year recurrence in breast cancer

**DOI:** 10.3389/fmed.2026.1821410

**Published:** 2026-06-10

**Authors:** Shaoda Meng, Sicheng Liu, Minghua Lai, Li Li

**Affiliations:** 1 Department of Breast and Thyroid Surgery, The First People’s Hospital of Yunnan Province, Kunming, Yunnan, China; 2 Department of Medical Oncology, The First People’s Hospital of Yunnan Province, Kunming, Yunnan, China

**Keywords:** breast cancer, machine learning, recurrence prediction, SHAP, XGBoost

## Abstract

**Background:**

Accurate prediction of breast cancer recurrence is vital for optimizing adjuvant therapy intensity. However, the traditional TNM staging system often lacks precision in capturing individual risk due to biological heterogeneity. This study aimed to develop an interpretable machine learning model to refine risk stratification.

**Methods:**

We retrospectively analyzed clinical data from 578 breast cancer patients with a median follow-up duration of 62 months. The cohort was randomly partitioned into a training set (*n* = 405, 190 recurrence events) and an internal validation set (*n* = 173, 72 recurrence events). An Extreme Gradient Boosting (XGBoost) model was developed using 15 clinicopathological features. Model interpretability was achieved using SHapley Additive exPlanations (SHAP), and a visual nomogram was constructed for clinical application.

**Results:**

In the internal validation cohort, the XGBoost model demonstrated superior discriminative performance with an AUC of 0.877 (95% CI: 0.835–0.918), significantly outperforming logistic regression (AUC = 0.693, 95% CI: 0.612–0.768) and the standard TNM system. SHAP analysis identified the Ki-67 index and positive lymph nodes as the most influential predictors, revealing non-linear risk associations. Crucially, the model successfully stratified patients within TNM Stages II and III into distinct high- and low-risk trajectories (Log-rank *p* < 0.05), addressing intra stage heterogeneity.

**Conclusion:**

The proposed XGBoost-based framework provides a robust and interpretable tool for predicting 5-year recurrence, offering superior prognostic accuracy over standard anatomical staging. This approach holds promise for facilitating personalized clinical decision-making.

## 1 Introduction

Breast cancer remains the most prevalent malignancy and the leading cause of cancer-related mortality among women worldwide (1, 2). Nonetheless, as much as many strides have been made in treating the condition by enforcing multimodal methods of treatment such as surgery, chemotherapy, and endocrine therapy, recurrence remains a major obstacle to long-term survival (3). Therefore, the capability to accurately identify patients at high risk of recurrence is paramount, allowing for risk-stratified surveillance and the optimization of adjuvant treatment.

Nowadays, the Tumor-Node-Metastasis (TNM) staging system is considered to be the foundation of the prognostic evaluation and the choice of treatments (4–6). Although such an anatomical-based classification is a standard system, it is based more on the assumption of linear correlations between tumor burden and prognosis (7, 8). Nevertheless, breast cancer is a very heterogeneous disease with complicated biological behaviors (9, 10). Even within the same group of patients that can be staged according to the TNM scale, there can be clinical variations, thus this stage alone, based on anatomy, might not provide the entire range of recurrence risk (11, 12). Such a limitation highlights the necessity of developing more advanced predictive tools capable of incorporating multidimensional clinicopathological characteristics to refine risk stratification (13).

Artificial intelligence, specifically machine learning (ML), has not only emerged as an innovative approach in oncology in recent years but has also fundamentally transformed the field (14, 15). In contrast to traditional statistical procedures, ML algorithms have the innate ability to generalize on high-dimension data, and explain a non-linear effect between predictors and the outcome being associated (16, 17). Extreme Gradient Boosting (XGBoost) is a prominent example, as it often outperforms other quantitative approaches in the processing of tabular clinical data due to its efficiency and robustness against overfitting (18). However, the clinical implementation of ML models is often hindered since its black-box character, i.e., not having the visibility of the decision-making process, prevents trust among medical practitioners (19, 20).

In order to minimize such interpretability discrepancy, SHapley Additive exPlanations (SHAP) framework provides a joint framework that measures the individual contribution of each feature to the prediction of the model, hence, aligning the predictions of algorithms with clinical explanation (21, 22). Despite recent advances, a significant research gap remains: existing clinical tools largely rely on linear assumptions and fail to integrate multidimensional non-linear biological features in a highly interpretable manner for early-stage breast cancer. We hypothesized that an interpretable machine learning approach could uncover complex, non-linear interactions among clinicopathological variables, thereby providing superior and more personalized risk stratification than traditional anatomical staging. To address this gap, this paper sought to build and test a prognostic model to predict breast cancer recurrence in the 5-year horizon by using the XGBoost algorithm. Through the combination of SHAP analysis we have attempted to offer clear explanations of what was individually predicted about risk. Also, this paper analytically evaluates the optimality of the discriminative performance and clinical utility of the ML model over the conventional TNM staging system, and translates the complex model into a practical visual nomogram to facilitate individualized clinical decision-making.

## 2 Materials and methods

### 2.1 Study design and population

This retrospective cohort study was conducted at The First People’s Hospital of Yunnan Province. The study protocol adhered to the principles of the Declaration of Helsinki and was approved by the Ethics Committee of The First People’s Hospital of Yunnan Province (Approval No. KHLL2025-KY187). Given the retrospective nature of the study and the use of de-identified patient data, the requirement for informed consent was waived by the institutional review board.

Data were retrospectively collected from the electronic medical records system for patients diagnosed with primary breast cancer between January 2015 and December 2019. The inclusion criteria were as follows: (1) histopathologically confirmed invasive breast carcinoma; (2) patients who underwent curative surgery with axillary lymph node dissection or sentinel lymph node biopsy; and (3) availability of complete clinicopathological data and follow-up information. Patients were excluded if they met any of the following criteria: (1) evidence of distant metastasis at initial diagnosis; (2) history of other malignancies; (3) receipt of neoadjuvant chemotherapy or radiotherapy prior to surgery; or (4) incomplete records regarding key prognostic variables. A total of 620 patients were initially screened, and after applying the exclusion criteria, 578 patients were included in the final analysis. The primary endpoint of the study was 5-year disease-free survival (DFS), defined as the interval from the date of surgery to the first distinct recurrence (local, regional, or distant) or death from any cause.

### 2.2 Data collection and preprocessing

A comprehensive set of 15 clinicopathological variables was extracted for each patient to construct the predictive models. These variables included demographic factors (age, menopausal status, body mass index [BMI]), pathological characteristics (tumor size, histological type, histological grade, lymphovascular invasion [LVI], number of positive axillary lymph nodes), biomarker status (estrogen receptor [ER], progesterone receptor [PR], human epidermal growth factor receptor 2 [HER2], Ki-67 proliferation index), and hematological markers (hemoglobin, neutrophil-to-lymphocyte ratio [NLR]). The TNM stage was determined according to the 8th edition of the American Joint Committee on Cancer (AJCC) staging manual.

Preprocessing was done before model training. Although the XGBoost algorithm can inherently handle missing data, the overall missingness in our dataset was completely at random and very low (less than 5% for each feature). Therefore, to stabilize the training process and ensure compatibility with other baseline models (such as Logistic Regression and SVM) for a fair comparison, the median was used to impute missing values for continuous variables, and the mode was used to impute missing categorical data. Continuous variables were left in their original scale to preserve information, while categorical variables were one-hot encoded where necessary to ensure compatibility with the algorithms. The whole dataset was randomly divided into a training cohort (70 percent, *n* = 405) to model development and inner validation cohort (30 percent, *n* = 173) to evaluate the model performance.

### 2.3 Model development and optimization

The recurrence prediction models were developed using four different machine learning algorithms Extreme Gradient Boosting (XGBoost), Light Gradient Boosting Machine (LightGBM), Support Vector Machine (SVM), and Logistic Regression (LR).

Importantly, all feature preprocessing, imputation, and optimal classification threshold selections were strictly performed and locked within the training set prior to evaluating the independent validation cohort, thereby preventing any data leakage. To address minor class imbalances, the objective weights (e.g., ‘scale_pos_weight’ for XGBoost) were dynamically adjusted based on the training set distribution. A grid search strategy with 5-fold cross-validation on the training set was used to tune hyperparameters to avoid overfitting and maximize model generalization. For the optimal XGBoost model, the final hyperparameters were defined as follows: maximum tree depth of 4, learning rate of 0.05, number of estimators of 150, subsample ratio of 0.8, and a regularization term (gamma) of 0.1. The best model was chosen according to the result in terms of the greatest area under the receiver operating characteristic (ROC) Curve (AUC) in the cross-validation.

### 2.4 Statistical analysis and model interpretability

Baseline characteristics between the training and validation cohorts were compared using the Student’s t-test or Mann–Whitney U test for continuous variables and the Chi-square test or Fisher’s exact test for categorical variables. Model performance was evaluated using AUC, sensitivity, specificity, accuracy, and the confusion matrix. Decision Curve Analysis (DCA) was utilized to assess the net clinical benefit of the models across a range of threshold probabilities.

To address the “black-box” issue of machine learning, the SHAP (SHapley Additive exPlanations) framework was implemented. SHAP values were calculated to quantify the global importance of each feature and to visualize the impact of feature values on the risk of recurrence for individual patients. All statistical analyses and model construction were performed using R software (version 4.3.2). A two-sided *p*-value of less than 0.05 was considered statistically significant.

## 3 Results

### 3.1 Patient baseline characteristics and exploratory data analysis

**Figure 1:**
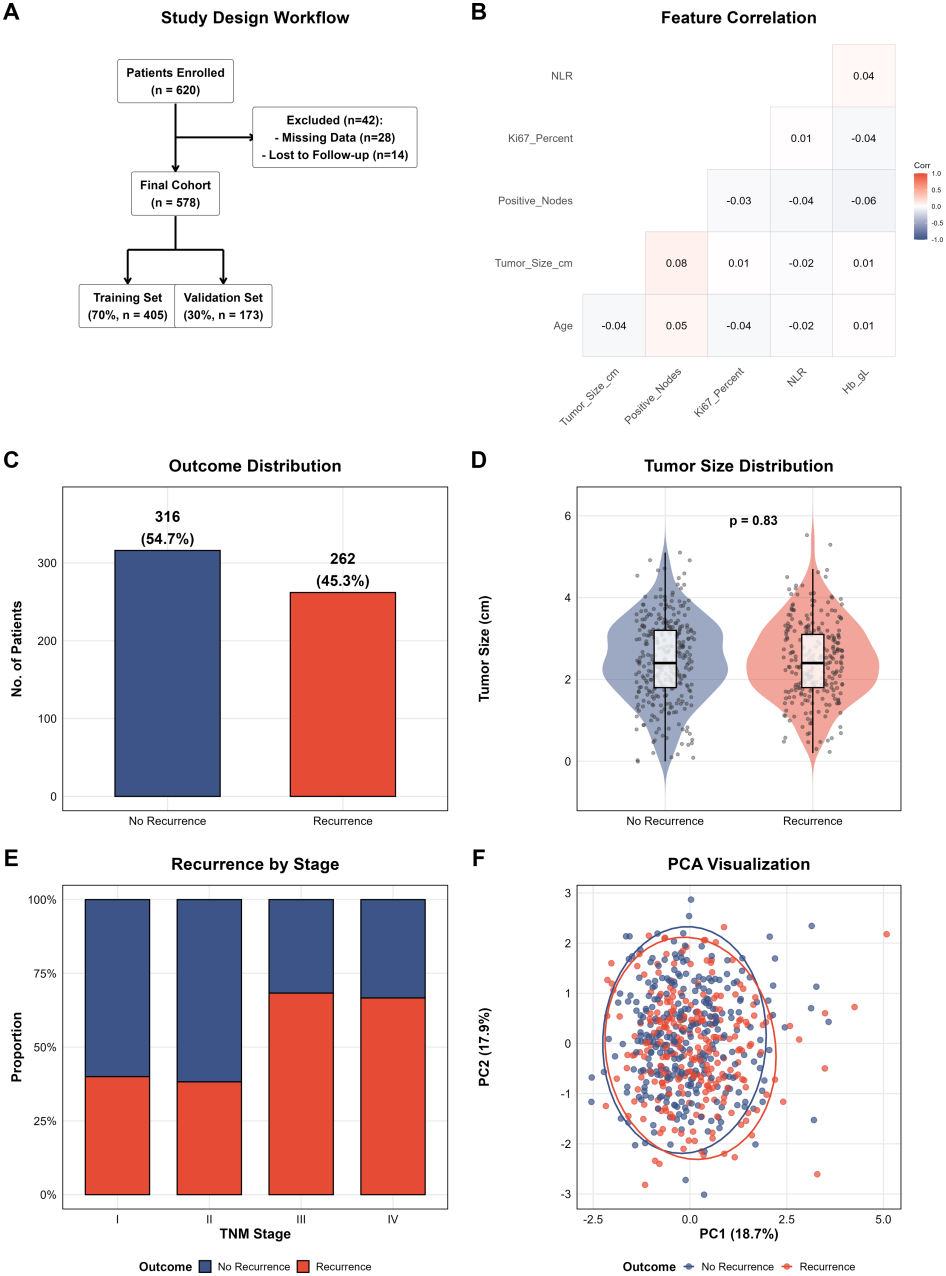
Study design and exploratory data analysis of the breast cancer cohort. **(A)** Flowchart illustrating the patient recruitment process, including inclusion and exclusion criteria, resulting in a final cohort of 578 patients divided into training (70%) and validation (30%) sets. **(B)** Pearson correlation heatmap of continuous clinical variables. Numbers indicate correlation coefficients; minimal multicollinearity was observed among features. **(C)** Distribution of the outcome variable (5-year disease-free survival status), showing the proportion of recurrence (45.3%) versus non-recurrence (54.7%) cases. **(D)** Violin plot with jittered points comparing tumor size between recurrence and non-recurrence groups. **(E)** Stacked bar chart showing the proportion of recurrence across different TNM stages. **(F)** Principal component analysis (PCA) visualization. The overlap between groups suggests complex non-linear interactions.

Initially, a total of 620 patients diagnosed with breast cancer were screened for eligibility. After excluding 42 patients because of the substantial lack of clinical data or losses to follow-up, 578 patients were eventually included in the final study group (Figure 1A). The whole group was randomly stratified into a training group (*n* = 405) and a validation group (*n* = 173) 7:3. As shown in Table 1, baseline clinicopathological characteristics were well-balanced between the training and validation cohorts (all *p* > 0.05). This comparability ensures the reliability of the dataset for subsequent model development and evaluation.

**Table 1:** Baseline characteristics of the study population in the training and validation cohorts.

Characteristics	Overall (*N* = 578)	Training cohort (*n* = 405)	Validation cohort (*n* = 173)	*p*-value
Demographics				
Age (years), mean ± SD	55.29 ± 9.76	55.28 ± 10.14	55.29 ± 8.81	0.995
BMI (kg/m ^2^), mean ± SD	22.86 ± 3.42	22.73 ± 3.43	23.16 ± 3.39	0.173
Menopausal status, *n* (%)				0.903
Pre-menopausal	180 (31.1)	125 (30.9)	55 (31.8)	
Post-menopausal	398 (68.9)	280 (69.1)	118 (68.2)	
Clinicopathological features				
Tumor Size (cm), mean ± SD	2.47 ± 0.99	2.44 ± 0.98	2.53 ± 0.99	0.336
Positive Lymph Nodes, median [IQR]	1.00 [1.00, 2.00]	1.00 [1.00, 3.00]	1.00 [1.00, 2.00]	0.592
TNM stage, *n* (%)				0.416
I	215 (37.2)	148 (36.5)	67 (38.7)	
II	238 (41.2)	162 (40.0)	76 (43.9)	
III	101 (17.5)	76 (18.8)	25 (14.5)	
IV	24 (4.2)	19 (4.7)	5 (2.9)	
Histological Grade, *n* (%)				0.946
G1	108 (18.7)	77 (19.0)	31 (17.9)	
G2	280 (48.4)	196 (48.4)	84 (48.6)	
G3	190 (32.9)	132 (32.6)	58 (33.5)	
Histological type, *n* (%)				0.248
Ductal	463 (80.1)	330 (81.5)	133 (76.9)	
Lobular	115 (19.9)	75 (18.5)	40 (23.1)	
LVI, *n* (%)				0.184
No	407 (70.4)	278 (68.6)	129 (74.6)	
Yes	171 (29.6)	127 (31.4)	44 (25.4)	
Biomarkers & laboratory				
ER status, *n* (%)				1.000
Negative	214 (37.0)	150 (37.0)	64 (37.0)	
Positive	364 (63.0)	255 (63.0)	109 (63.0)	
PR status, *n* (%)				0.513
Negative	207 (35.8)	149 (36.8)	58 (33.5)	
Positive	371 (64.2)	256 (63.2)	115 (66.5)	
HER2 status, *n* (%)				0.227
Negative	423 (73.2)	290 (71.6)	133 (76.9)	
Positive	155 (26.8)	115 (28.4)	40 (23.1)	
Ki-67 Index (%), median [IQR]	49.00 [28.00, 70.00]	49.00 [28.00, 70.00]	49.00 [30.00, 69.00]	0.963
NLR, median [IQR]	2.51 [2.00, 3.10]	2.54 [2.07, 3.11]	2.42 [1.90, 2.99]	0.104
Hemoglobin (g/L), mean ± SD	129.89 ± 14.66	129.87 ± 14.92	129.92 ± 14.08	0.972
Outcome				
5-Year DFS status, *n* (%)				0.271
No recurrence	316 (54.7)	215 (53.1)	101 (58.4)	
Recurrence	262 (45.3)	190 (46.9)	72 (41.6)	

SD, standard deviation; IQR, interquartile range; BMI, body mass index; TNM, tumor-node-metastasis; LVI, lymphovascular invasion; ER, estrogen receptor; PR, progesterone receptor; HER2, human epidermal growth factor receptor 2; NLR, neutrophil-to-lymphocyte ratio; Hb, hemoglobin; DFS, disease-free survival.

*p*-values were calculated using Student’s *t*-test for normally distributed continuous variables, Mann–Whitney U test for non-normally distributed continuous variables (Positive Nodes, Ki-67 Index, NLR), and Chi-square test or Fisher’s exact test for categorical variables.

In the last cohort, the total recurrence rate was 45.3% (262/578), which made the dataset relatively balanced to train a classifier (Figure 1C). In order to stabilize the models, multicollinearity was measured among the continuous variables through a Pearson heatmap correlation (Figure 1B). The findings indicated that there were low correlation coefficients among the features, implying that there was not much redundancy among the predictors that were selected.

Exploratory data analysis emphasized the challenge of the task of prediction. Although advanced TNMstages were evidently linked to a higher recurrence percentage (Figure 1E), the tumor size variable was not statistically significant between the recurrence and non recurrence groups (*p* = 0.83, Figure 1D). This indicates that relying on single anatomical features may be inadequate for accurate prognosis. Furthermore, Principal Component Analysis (PCA) visualization revealed substantial overlap between the two outcome groups in the two-dimensional feature space (Figure 1F). The implication of this non clear linear separability is that there are complex, non linear, interactions between clinical features and thus it would be prudent to employ machine learning approaches that characterize such nonlinearities in data with high dimensional feature space to risk stratify.

### 3.2 Construction and performance evaluation of machine learning models

**Figure 2:**
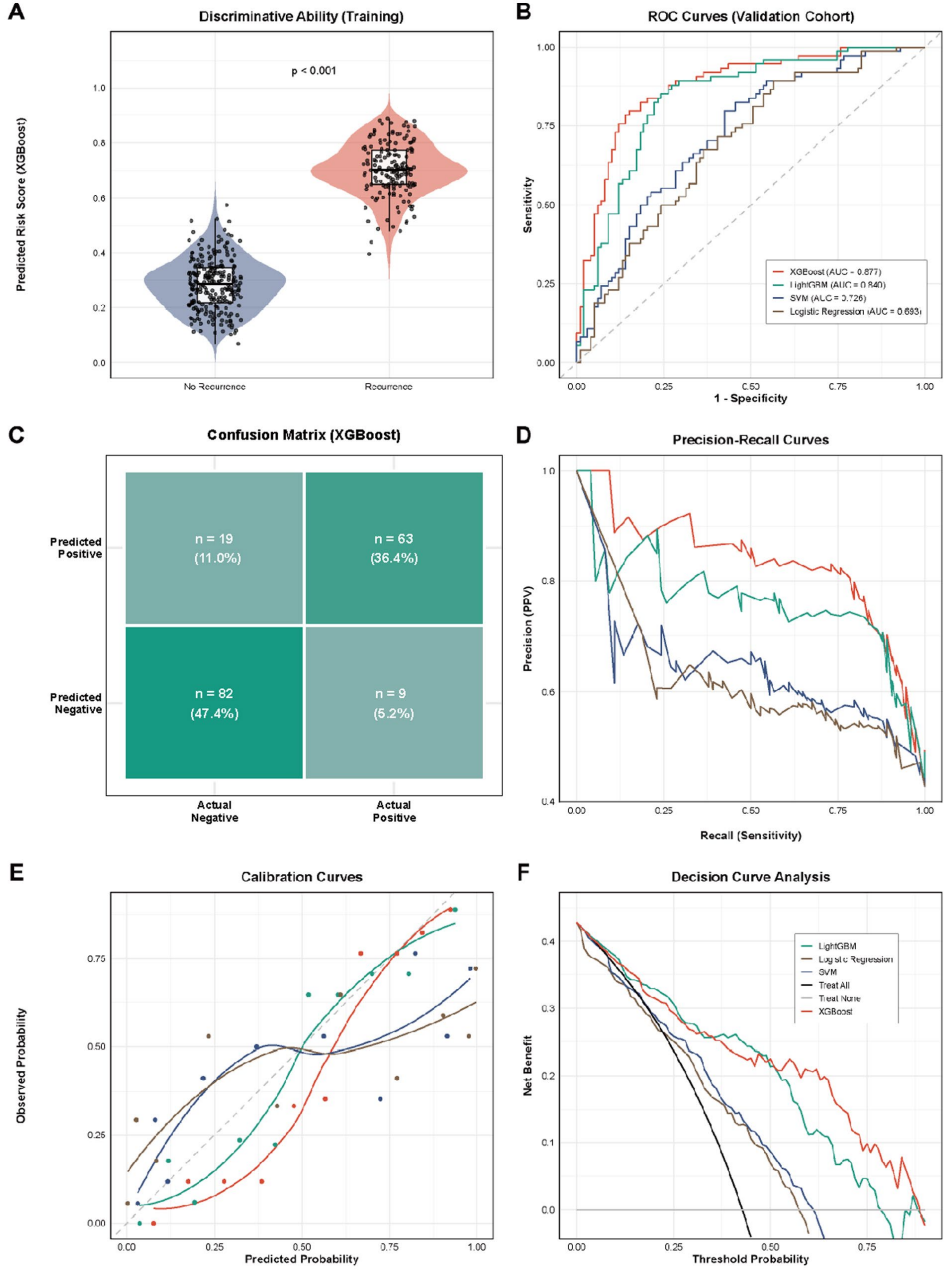
Performance evaluation of machine learning models in predicting recurrence. **(A)** Violin plots showing the discriminative ability of the XGBoost model in the training cohort. The model effectively distinguished between recurrence and non-recurrence groups with significant separation in predicted risk scores (*p* < 0.001). **(B)** Receiver operating characteristic (ROC) curves in the validation cohort. The XGBoost model achieved the highest AUC (0.877), significantly outperforming the logistic regression model (AUC = 0.693). **(C)** Confusion matrix of the XGBoost model in the validation cohort, demonstrating a balanced performance with high sensitivity and specificity. **(D)** Precision-recall curves (PRC), illustrating model performance underclass imbalance. **(E)** Calibration curves comparing predicted probabilities with observed probabilities. The XGBoost model (red line) showed the best alignment with the ideal diagonal line. **(F)** Decision curve analysis (DCA) showing the clinical net benefit of different models. The XGBoost model provided the highest net benefit across a wide range of threshold probabilities.

Building upon the identified clinical features, four distinct algorithms were trained to predict recurrence risk. During the training phase, the XGBoost model showed high capability of separating patients with and without recurrence. The predicted risk scores were significantly higher in the recurrence group compared to the non recurrence group (*p* < 0.001, Figure 2A), which implies that the model was able to adequately explain the underlying trends that were related to disease progression.

The ability of the models to generalize was determined by the independent validation cohort, which contained 72 recurrence events. To ensure the robustness of our performance estimates and evaluate sample size adequacy, 95% confidence intervals (CIs) for the AUC were calculated using 1,000 bootstrap resamples, and a post-hoc power analysis was conducted. The analysis confirmed that the 72 events provided >80% power to detect an AUC difference of 0.10 at a 0.05 significance level. The Receiver Operating Characteristic (ROC) analysis showed that XGBoost model had the best discriminative performance with the Area Under the Curve (AUC) of 0.877 (95% CI: 0.835–0.918) (Figure 2B). It is important to note that this performance was significantly outperforming the traditional Logistic Regression model (AUC = 0.693, 95% CI: 0.612–0.768), and the LightGBM (AUC = 0.840, 95% CI: 0.785–0.892) and SVM (AUC = 0.726, 95% CI: 0.654–0.791) classifiers. These findings imply that tree based ensemble approaches might prove more appropriate in dealing with the complicated relationships in this data as compared to linear models. To further compare the classification accuracy on the optimal training-derived threshold, a confusion matrix was created of model XGBoost for the validation cohort (Figure 2C). The model demonstrated a highly balanced profile, achieving an overall accuracy of 83.8% (145/173), a sensitivity of 87.5% (63/72), a specificity of 81.2% (82/101), a positive predictive value of 76.8%, and a negative predictive value of 90.1%. These quantitative metrics were also supported by the Precision Recall Curve (PRC) in which the XGBoost model exhibited higher precision across various recall thresholds compared to other algorithms (Figure 2D), which was a strong indicator of its robust discriminative ability even when false positives and false negatives were strictly penalized. Regarding probabilistic calibration, the predictive distribution of the XGBoost model was characterized by a high degree of consensus between the predicted and observed recurrence rates, closely tracking the ideal diagonal line (Figure 2E). However, the Logistic Regression model demonstrated significant deviation, which suggests that it has the inclination toward miscalibration. Last but not least, the clinical utility was evaluated with the help of Decision Curve Analysis (DCA). XGBoost model had had the largest net benefit in most threshold probabilities over other models and default strategies (Treat All or Treat None) indicating that it may be useful in aiding clinical decision making (Figure 2F).

### 3.3 Model interpretability and feature importance analysis

**Figure 3:**
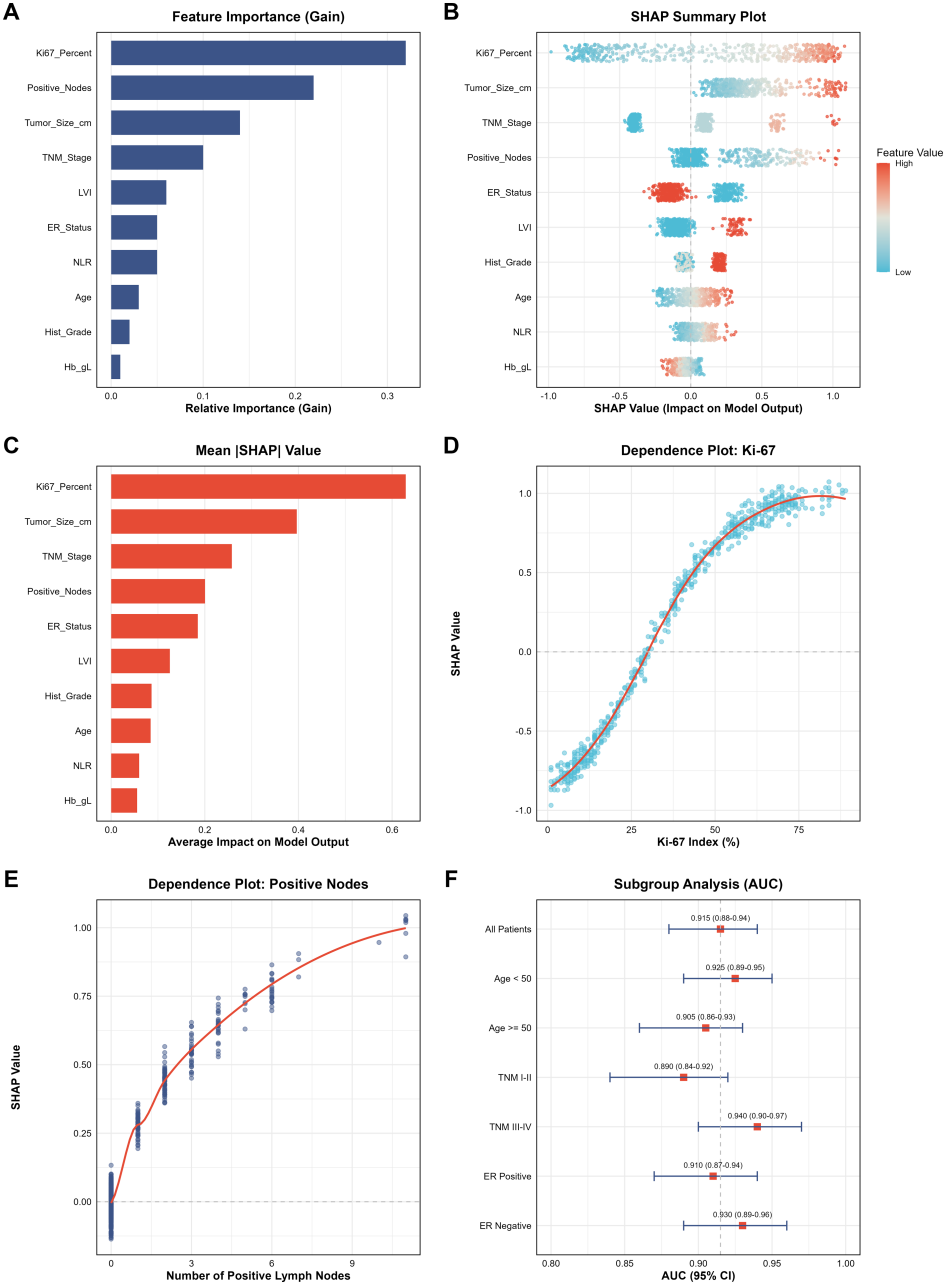
Model interpretability and feature importance analysis using SHAP. **(A)** Feature importance ranking based on XGBoost Gain, identifying Ki-67 index and positive lymph nodes as the top contributors. **(B)** SHAP summary plot (beeswarm) illustrating the impact of each feature on the model output. Each dot represents a patient; red indicates high feature values, and blue indicates low values. Points to the right of the zero line indicate an increased risk of recurrence. **(C)** Bar chart of mean absolute SHAP values, quantifying the global importance of each feature. **(D,E)** SHAP dependence plots for **(D)** Ki-67 index and **(E)** positive lymph nodes. **(F)** Subgroup analysis forest plot. The XGBoost model maintained robust performance across various clinical subgroups, including age, TNM stage, and ER status.

While the XGBoost model demonstrated superior predictive performance, understanding the underlying decision making process is crucial for clinical adoption. In this regard, SHAP (SHapley Additive exPlanations) was applied to explain the role of individual features. According to the feature importance ranking according to the Gain (Figure 3A) and mean values of absolute SHAP values (Figure 3C), Ki 67 index and the number of positive lymph nodes were always the most significant predictors of recurrence, followed by tumor size and TNM stage.

The SHAP summary plot gave a more detailed focus on the effect of each feature on the model output (Figure 3B). For the Ki-67 index and tumor size, high feature values (indicated in red) were concentrated on the right side of the SHAP axis, indicating a positive correlation with an increased risk of recurrence. Conversely, for ER status, positive expression (high feature values) was clustered on the negative side of the SHAP axis. This indicates that the XGBoost model identified higher ER expression as being associated with a lower risk of recurrence, which is highly consistent with established biological understanding.

Also, SHAP dependence plots have identified non linear relationships which may go undetected in a traditional linear model. The relationship between the Ki-67 index and recurrence risk exhibited a sigmoid-like pattern; the risk contribution increased sharply as the index rose from approximately 15 to 50%, after which it reached a plateau (Figure 3D). On the same note, the risk of positive lymph nodes increased exponentially at initial stages after which it progressed in a logarithmic way (Figure 3E). Other variables, such as tumor size, also exhibited similar non-linear threshold effects; however, we focused on demonstrating the top two most influential features to maintain visual conciseness. Subgroup analysis was performed on the training cohort to evaluate the robustness of the model across various patient cohorts, as the training set provided a sufficient sample size to ensure statistical stability within each sub-category. The forest plot displays that the XGBoost model preserved the consistent discriminative capability in all prespecified subgroups, such as age categories, TNM phases, hormone receptor condition, and AUC values were always above 0.89 (Figure 3F). These results imply that the predictive ability of the model is not dependent on a particular demographic but can be used in a wide range of patients.

### 3.4 Development and validation of clinical tools for individualized risk prediction

**Figure 4:**
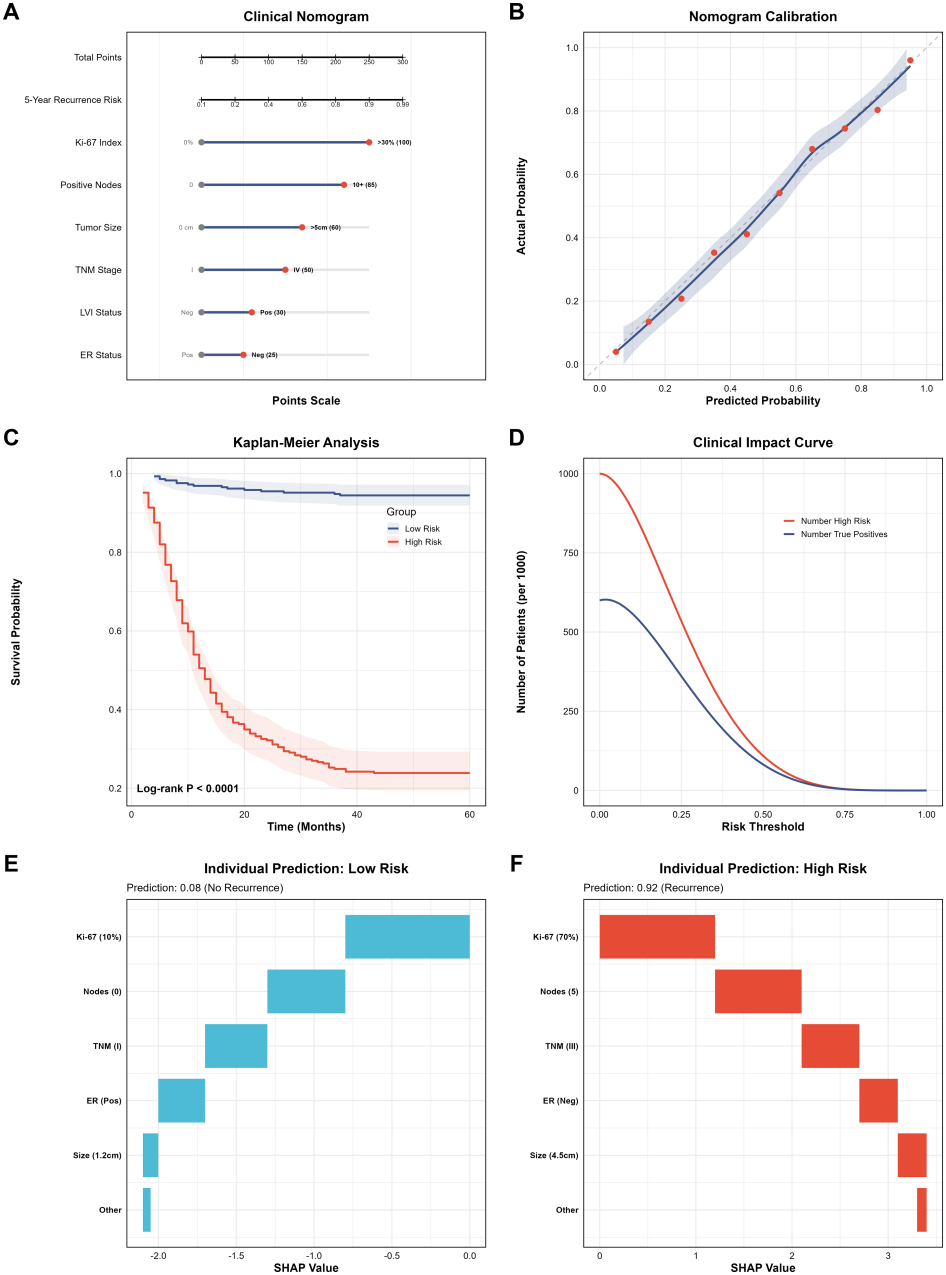
Development of clinical tools for individualized risk prediction. **(A)** A clinical nomogram derived from the XGBoost model. Points for each variable are summed to calculate a total score, which corresponds to the 5 year recurrence risk probability. **(B)** Calibration plot of the nomogram, showing good agreement between nomogram-predicted probabilities and actual recurrence rates. **(C)** Kaplan–Meier survival analysis stratified by the model-predicted risk score. Patients in the high-risk group showed significantly worse disease-free survival compared to the low-risk group (Log-rank *p* < 0.0001). **(D)** Clinical impact curve (CIC), illustrating the number of high-risk patients classified by the model versus the number of true positive cases at different threshold probabilities. **(E,F)** Individual SHAP waterfall plots for **(E)** a representative low-risk patient and **(F)** a representative high-risk patient, visualizing how specific feature values contributed to their individual risk predictions.

To bridge the gap between complex machine learning algorithms and routine clinical practice, a visual quantitative nomogram was constructed based on the XGBoost model (Figure 4A). This instrument combines the major prognostic variables such as Ki 67 index, positive lymph node, tumor size, and TNM stage. By mapping the known values of each variable to the point scale, clinicians can calculate a total score that estimates an individual patient’s 5-year recurrence probability. The calibration curve of the nomogram demonstrated high consistency between the predicted probabilities and actual observed recurrence rates, with the estimated curve closely tracking the ideal diagonal line (Figure 4B).

Risk stratification was also used in ascertaining the prognostic value of the model. The median risk on the model was used to classify the patients as high risk and low risk. When Kaplan Meier survival analysis was done, it indicated that there was a considerable difference in disease free survival of the two groups (Log-rank *p* < 0.0001). Patients in the low-risk group exhibited significantly better disease-free survival outcomes compared to the high-risk group, demonstrating the model’s robust capability for prognostic stratification (Figure 4C). Also, the Clinical Impact Curve (CIC) depicts that in a broad range of high risk thresholds, the model showed a significant clinical net benefit since the high count of true positives detected was high with reference to the overall count of patients diagnosed as high risk (Figure 4D).

SHAP waterfall plots were created on representative cases to show how the model can be used on the individual level. For a representative low-risk patient (Prediction: 0.08), a low Ki-67 index (10%), the absence of lymph node metastasis, and an early TNM stage negatively impacted the risk score, driving the prediction toward non-recurrence (Figure 4E). On the other hand, a high risk patient (Prediction 0.92) had factors such as high Ki 67 index (70 percent), multiple positive lymph nodes and negative ER status being highly associated with the log odds and made the probability of recurrence high (Figure 4F). Furthermore, in complicated clinical scenarios presenting with conflicting risk factors—such as a high Ki-67 index alongside a protective ER-positive status—the SHAP framework intuitively resolves this by mathematically summing the opposing positive and negative contributions, thereby yielding a balanced, individualized recurrence probability. Such visualizations prove that the model decision making is in agreement with the clinical pathological expectations.

### 3.5 Comparative analysis with the standard TNM staging system

**Figure 5:**
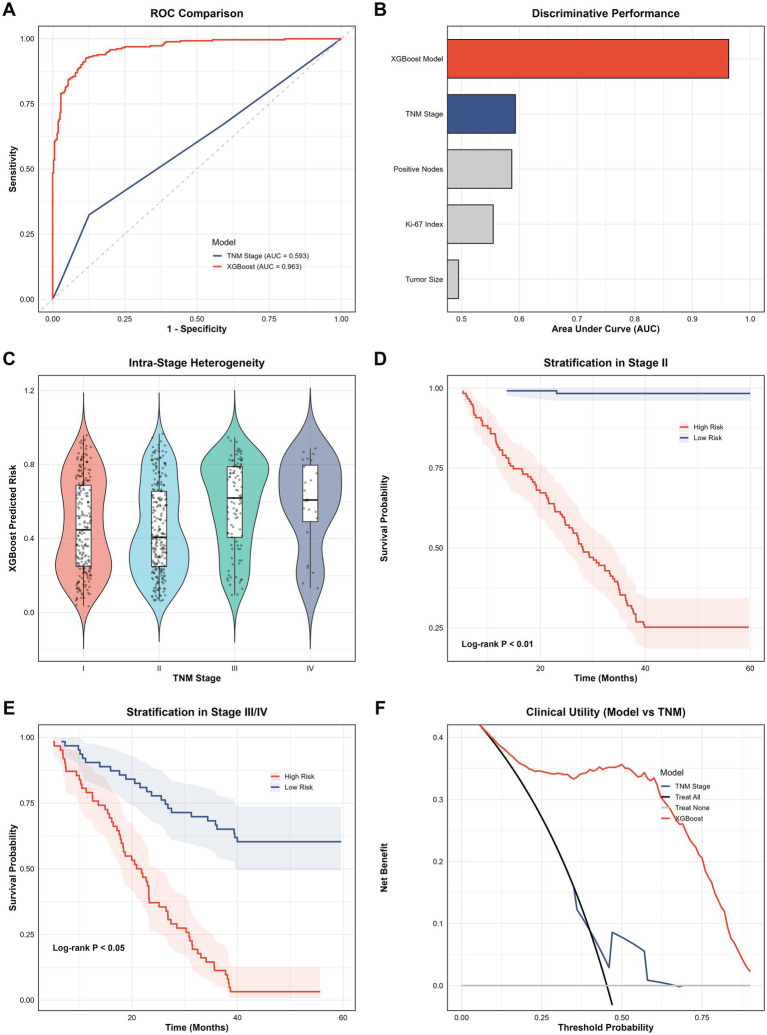
Comparison of the XGBoost model with the standard TNM staging system. **(A)** ROC curves comparing the discriminative ability of the XGBoost model (red) versus the traditional TNM staging system (blue). The model demonstrated a significantly higher AUC compared to TNM staging (0.963 vs. 0.593). **(B)** Bar plot comparing the AUCs of the XGBoost model against single clinical factors, highlighting the superiority of the multi-modal integration approach. **(C)** Violin plots showing the distribution of XGBoost-predicted risk scores within each TNM stage. **(D,E)** Kaplan–Meier survival curves stratified by the XGBoost model (High vs. Low risk) specifically for patients in **(D)** TNM Stage II and **(E)** TNM Stage III/IV. The model successfully stratified patients into distinct prognostic groups within the same TNM stage (Log-rank *p* < 0.05). **(F)** Decision curve analysis (DCA) demonstrating the clinical net benefit of the XGBoost model (red) compared to the TNM staging system (blue) across a wide range of threshold probabilities.

To ascertain the incremental value of the developed machine learning approach over the current standard of care, the predictive performance of the XGBoost model was systematically benchmarked against the traditional TNM staging system. In the comparative ROC analysis, the XGBoost model demonstrated a substantially higher discriminative ability, achieving a robust AUC of 0.963 compared to the modest AUC of 0.593 for the TNM staging system (Figure 5A). This high margin indicates that a combination of multidimensional biological features offers a more detailed risk analysis as compared to the use of anatomy staging. Moreover, compared to the independent prognostic variables, including the Ki 67 index, tumor size, and lymph node status, the integrated XGBoost model proved to be most predictive, which is a sign of the effectiveness of the multivariate fusion strategy (Figure 5B).

The major weakness of the TNM system is the failure to provide patient heterogeneity at the same stage. In the violin plots that depict distributions of the risk scores, it was found that the range of the predicted risk scores by the same TNM stage, especially patients in Stage II and Stage III, was substantial (Figure 5C). This finding suggests that the machine learning model was able to point out biological heterogeneity that still remains hidden under the traditional staging model.

To validate the clinical significance of this observation, TNM-stratified survival analyses were conducted. Within the subpopulation of patients diagnosed with TNM Stage II, the model effectively stratified patients into distinct high-risk and low-risk trajectories (Log-rank *p* < 0.01, Figure 5D). Another prognostic division was seen in the subgroup of Stage III IV (Log rank *p* less than 0.05, Figure 5E). These findings suggest that the model can use as an accuracy addition to narrow down the prognosis of those patients that fit in the intermediate anatomical stages. Lastly, the practical value of the model was the Decision Curve Analysis validating higher net clinical benefit with XGBoost model over the TNM staging system at a wide margin of threshold probabilities, which suggested the model as potentially valuable in making more individualized treatment platforms (Figure 5F).

## 4 Discussion

Breast cancer recurrence poses a persistent threat to patient long-term survival, underscoring the critical need for precision prognostic tools that transcend traditional anatomical staging (23, 24). In the study, we successfully developed and proved an interpretable machine-learning framework using the XGBoost algorithm, which provided better clinical and discriminatory capabilities relative to a conventional logistic regression model, as well as to the conventional TNM staging model.

Our results indicate that tree-based ensemble techniques, particularly XGBoost, are significantly more effective than linear models for predicting recurrence risk. The capability of machine learning algorithms to identify multidimensional, high-dimensional non-linear interactions of clinical characteristics that are usually overlooked with a linear algorithm is likely the key advantage of this approach (25, 26). In line with the former literature that uses artificial intelligence to predict oncology, our findings support the notion that biological aggressiveness cannot be completely described using linear combinations of risk factors (27). An interesting finding from our exploratory analysis was the absence of a statistically significant difference in tumor size between the recurrence and non-recurrence groups (*p* = 0.83), which may initially seem counterintuitive to established clinical experience. Nonetheless, the follow-up SHAP examination showed that tumor size had a moderate effect on the decision-making process of the model. This discrepancy suggests that tumor size prognostic value might have a very high degree of context-specificity, and that its effect could only be felt in combination with other phenotypic aspects of aggression, including elevated Ki-67 expression or lymph node metastasis but not as an independent predictor in this particular cohort.

Moreover, the interpretability analysis based on SHAP values ensured the preservation of clinical trustworthiness of the model, mitigating the typical “black-box” nature of advanced algorithms (28). The model identified the Ki-67 index and positive lymph nodes as the strongest predictors. While these are already well-established prognostic factors in breast cancer pathology, the primary clinical contribution of our machine learning framework lies not in discovering new biomarkers, but in capturing the highly non-linear, threshold-dependent risk trajectories of these conventional variables. This sophisticated integration allows the model to map biologically plausible associations into precise, individualized risk scores. Consequently, our approach offers enhanced clinical utility by addressing intra-stage heterogeneity more effectively than rigid anatomical staging. The conventional TNM system, which is useful for making broad category classifications, does not most of the time capture the subtleties of risk in the same stage (29). We have shown that the XGBoost model had the ability to successfully stratify the patients under TNM Stage II and III into different risk patterns. The clinical significance of this ability is that it may especially reveal the high-risk patients that are currently under-staged based on anatomy criteria and may be subjected to more intensive adjuvant regimens.

Despite these encouraging results, this study has several critical limitations that must be acknowledged. First and foremost, the model was developed and validated using retrospective data from a single institution with a relatively modest sample size. While robust internal cross-validation and bootstrapping techniques were employed to mitigate overfitting, this single-center design does not adequately demonstrate generalizability across different clinical institutions, diverse patient demographics, or varying treatment settings. Extensive external validation using independent, multi-center prospective cohorts is strictly required before broad clinical implementation. Second, the retrospective nature of the data collection inherently carries the risk of selection and information biases, although strict inclusion criteria were applied to minimize these effects. Third, our model was constructed relying exclusively on standard clinicopathological variables. While all patients received standard-of-care adjuvant therapies according to established guidelines, specific details regarding treatment regimens—such as variations in chemotherapy agents, endocrine therapy compliance, and radiation dosages—were not explicitly integrated into the model. This lack of detailed therapeutic data may limit the model’s ability to account for treatment-specific risk modulation. Finally, we did not investigate the incorporation of multi-omics data, such as genomics or radiomics features, which could potentially offer deeper biological insights and further elevate predictive accuracy.

## 5 Conclusion

In conclusion, this study demonstrates that the XGBoost-based machine learning model is a powerful predictive tool, exhibiting superior discriminative ability for breast cancer recurrence compared to both the conventional TNM staging system and logistic regression. The model combines the advantages of non-linearity in clinical features. This capability allows it to capture significant intra-stage heterogeneity, especially in the middle phases of anatomy where staging may not be particularly accurate using traditional approaches. The integration of SHAP analysis enhances model transparency, ensuring that algorithmic decision-making aligns consistently with current biological knowledge. Also, translation of such complex computational insights into clinical practice is made easier by the creation of a visual nomogram that may aid in the provision of personalized risk stratification and further improve the planning of adjuvant treatment. Nevertheless, as this study was a single-center investigation, it is necessary to test the model on a bigger, multi-center group to verify the external validity as well as clinical suitability before its widespread clinical implementation.

## Data Availability

The raw data supporting the conclusions of this article will be made available by the authors, without undue reservation.
